# Dose escalating study of cetuximab and 5-FU/folinic acid (FA)/oxaliplatin/irinotecan (FOLFOXIRI) in first line therapy of patients with metastatic colorectal cancer

**DOI:** 10.1186/1471-2407-14-521

**Published:** 2014-07-19

**Authors:** Gunnar Folprecht, Susanne Hamann, Katharina Schütte, Tanja Trarbach, Jan Stoehlmacher-Williams, Gerhard Ehninger

**Affiliations:** 1 Medical Department I, University Hospital Carl Gustav Carus, University Cancer Center, Fetscherstr 74, 01307 Dresden, Germany; 2 Medical Department IV, Klinikum Chemnitz, Chemnitz, Germany; 3 Onkologische Gemeinschaftspraxis, Arnoldstr. 18, Dresden, Germany; 4 Medical Department/ Tumour research, West Germany Cancer Center, Essen, Germany; 5 ioMedico, Freiburg, Germany; 6 Onkologische Praxis, Bonn, Germany

**Keywords:** Cetuximab, Phase I, Metastatic colorectal cancer, Irinotecan, Oxaliplatin, 5-FU, Chemotherapy

## Abstract

**Background:**

The FOLFOXIRI regimen (irinotecan, oxaliplatin, fluorouracil [5-FU] and folinic acid [FA]) increased the response rate and overall survival compared to FOLFIRI in patients with metastatic colorectal cancer (mCRC). Adding cetuximab to FOLFOX or FOLFIRI increased efficacy in patients with k-ras wild type mCRC. We explored the dose limiting toxicity and feasibility of the combination cetuximab, irinotecan, oxaliplatin, 5-FU and FA in mCRC patients.

**Methods:**

In a dose-escalation study patients with previously untreated mCRC and a WHO performance status 0–1 received cetuximab (500 mg/m^2^, 2 h), followed by irinotecan (95, 125, and 165 mg/m^2^ in the dose levels [DL] 1, 2, and 3 respectively), followed by oxaliplatin (85 mg/m^2^, 2 h) which was given parallel to FA (400 mg/m^2^, 2 h) and followed by 5-FU (3200 mg/m^2^, 46 h) in an outpatient setting every two weeks. The primary endpoints were the maximum tolerable dose and the safety. The trial was approved by the local ethics committee.

**Results:**

From 2007 to 2008, twenty patients were treated in this trial. In the first dose level (irinotecan 95 mg/m^2^) one patient developed neutropenia grade 4. One patient experienced diarrhoea grade 3 as DLT in dose level 2 (irinotecan 125 mg/m^2^). In dose level 3 (irinotecan 165 mg/m^2^), three patients experienced a DLT (diarrhoea grade 3 and two patients with neutropenia grade 4). Thus, the recommended dose for a phase II trial is 125 mg/m^2^ irinotecan in combination with oxaliplatin, 5-FU/FA and cetuximab. Most common grade ≥3 toxicities were neutropenia (40%), diarrhoea (25%) and acne-like rash (15%). No therapy associated death occurred.

The confirmed overall response rate in all cohorts was 75% (95%-CI 51-91%). The best response was reached after a median of 3.0 (95%-CI 2.2 to 3.7) months. Median progression free survival (PFS) is 16 (95%-CI 12.6-19.4) months, overall survival (OS) 33 (95%-CI 26.2-39.8) months.

**Conclusions:**

The combination of cetuximab and FOLFOXIRIis feasible and has an acceptable toxicity profile in patients with a good performance status. The observed clinical activity with a confirmed response rate of 75% is promising and further evaluated in the ongoing CELIM2.

**Trial registration:**

http://www.clinicaltrials.gov: NCT00422773.

## Background

Colorectal cancer (CRC) is a leading cause of cancer related death in Europe. Despite advances in the systemic treatment of metastatic colorectal cancer (mCRC), the long term prognosis of patients with (mCRC) still remains unfavourable unless a resection can be performed. Metastasectomies can provide curative treatment with a 5-year overall survival of 48% if (liver) metastases are resectable at the time of diagnosis of the metastases and 33%, if initially non-resectable liver metastases are resected after tumour shrinkage due to chemotherapy [1]. Therefore, a major treatment goal of initially unresectable mCRC is to intensify chemotherapy to increase the number of secondarily resectable patients. As there is a correlation between response to chemotherapy and the resection rate [2] it might be interesting to develop chemotherapy schedules that are able to induce early and meaningful tumour shrinkage.

Both irinotecan and oxaliplatin, each in combination with infusional 5-fluorouracil (5-FU)/ folinic acid (FA) have been the standard chemotherapy regimens in mCRC inducing response rates (RR) of 40% to 50% [3,4]. One strategy to maximize tumour response is to combine all cytotoxic drugs into a “FOLFOXIRI” regimen. Such an approach had promising results in earlier phase II trials [5]. An Italian phase III trial demonstrated an increase of the RR and a prolongation of the progression free and overall survival with significantly more liver resections in the FOLFOXIRI arm, but also increased toxicity, especially diarrhoea and neutropenia compared to the FOLFIRI arm [6].

Cetuximab improved efficacy when combined with oxaliplatin/5-FU/FA [7] or with irinotecan/5-FU/FA if activating k-ras mutations were absent. With cetuximab plus FOLFIRI, the rate of liver resections was increased compared to FOLFIRI alone [8]. Although the typical toxicities of cetuximab monotherapy are mostly limited skin and allergic reactions [9], diarrhoea and other toxicities were increased if cetuximab was combined with chemotherapy [7,10].

The current study was carried out to determine a dose of the combination of cetuximab, irinotecan, oxaliplatin and 5-FU/FA for further evaluation in neoadjuvant treatment of patients with colorectal liver metastases in future trials.

## Methods

### Study design

This open-label, non-randomized, dose-escalation trial was planned to determine the MTD for FOLFOXIRI and Cetuximab in the first line treatment of patients with metastatic colorectal cancer. It was conducted in two German University Cancer Centres.

The primary objective was to assess a maximum tolerable dose and the safety of the chemotherapy-antibody-combination of cetuximab, irinotecan, oxaliplatin and 5-FU/folinic acid as first-line treatment for metastatic colorectal cancer. Secondary objectives were feasibility, toxicity, response rate, resection rate, progression free and overall survival.

Three or six patients were planned to receive chemotherapy at each dose level starting with the lowest dose-level. The dose was escalated by one dose level if none of the first three patients or less than two of six patients at a particular dose level experienced a dose-limiting toxicity (DLT) during the first six weeks.

Up to four dose levels and a MTD cohort of 16 patients to explore the irinotecan dosing schedule in the combination of FOLFOXIRI/cetuximab were originally planned. However, this extended phase was not effected as the recruitment was stopped when the influence of the k-ras mutational status in first line therapy was demonstrated [8].

### Patient selection

Patients with histologically confirmed non-resectable colorectal cancer, without prior treatment for metastatic disease and with a WHO performance status (PS) 0–1 were eligible for this trial. They were enrolled 2007/2008 and were not selected for epidermal growth factor receptor (EGFR) immunohistochemistry or k-ras status.

Exclusion criteria were prior anti- EGFR therapy, prior radiotherapy or major abdominal or thoracic surgery within the last 4 weeks before inclusion, known hypersensitivity reaction to any of the components of the study treatment, clinically relevant coronary disease or myocardial infarction within 12 months before study entry, peripheral neuropathy Common Terminology Criteria for Adverse Events (CTCAE) > grade I, acute or sub-acute intestinal obstruction or inflammatory bowel disease, previous malignancy (except CRC, history of basal cell carcinoma of skin or pre-invasive carcinoma of the cervix with adequate treatment), brain metastasis, adjuvant treatment within 6 months before study, inadequate renal, hepatic or hematologic function, history of severe psychiatric illness, concurrent anti-cancer treatment, age <18 years and pregnant or lactating women.

### Treatment and evaluation

Patients received biweekly i.v. doses of cetuximab (500 mg/m^2^, 2 h), followed by irinotecan (95, 125, and 165 mg/m^2^ in the dose levels [DL] 1, 2, and 3), followed by oxaliplatin (85 mg/m^2^, 2 h) which was given parallel (via separate line) to FA (400 mg/m^2^, 2 h) and followed by 5-FU (3200 mg/m^2^, 46 h) in an outpatient setting. Twelve cycles were planned.

Adverse events (AE) were rated according to the National Cancer Institute-Common Toxicity Criteria (NCT-CTC, version 3.0).

DLTs were defined as: neutropenia grade 4 or febrile neutropenia, thrombocytopenia grade 3, diarrhoea grade 3 lasting > 24 h if adequately treated with loperamide or diarrhoea grade 3 in combination with neutropenia grade ≥ 3, nausea/ vomiting grade 3 despite anti-emetic treatment, acne-like rash grade 4 and other non–haematological grade 3 toxicity (except alopecia). Hematopoietic growth factors were allowed after neutropenia grade 3.

Imaging for the assessment of tumour response was carried out at baseline (within three weeks before treatment start) and repeated after every third cycle (6 weeks). Responses were assessed and confirmed according to RECIST1.0 [11]. A waterfall plot was calculated by the percentage of tumour reduction between baseline and time of best response for measurable target lesions.

Progression free survival and overall survival were calculated by the method of Kaplan-Meier. Patients who started a subsequent therapy line without formal progression were censored for the calculation of progression free survival.

K-ras was retrospectively analysed for mutations in codon 12/13 after macrodissection by pyrosequencing using the commercial therascreen kit (Qiagen). The dose intensity per cycle was calculated as the relationship of delivered by the planned doses from treatment start multiplied by the ratio of planned vs actual treatment days.

### Ethical considerations and funding

All patients gave written informed consent. The trial was conducted according to the Declaration of Helsinki with amendments and the Guidelines for Good Clinical Practice ICH Tripartite Guideline. It was approved by the local ethics committee and the national and federal health authorities.

The legal sponsor of this trial was the Technical University of Dresden which had the full control of the data base.

The study was funded by educational grants from Merck Pharma Deutschland GmbH and Pfizer Pharma Deutschland GmbH.

## Results

### Patients

**Figure 1:**
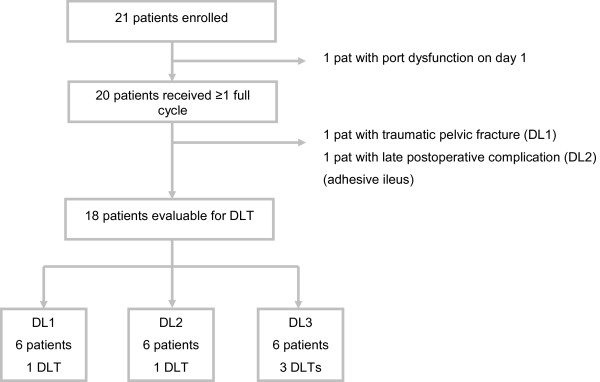
Contribution of patients on the separate dose levels.

Twenty-one patients were enrolled into the study between January 2007 and June 2008. One patient registered for DL2 was excluded from further treatment and all analyses because of leakage of his i.v. port-catheter system before the first full dose of chemotherapy was completed (Figure 1).

All analyses refer to the remaining 20 patients who actually received at least one full cycle of study treatment. Median age was 59 (34–72) years. Fifteen patients presented with a WHO PS 0 (75%), fifteen were male (75%). Ten patients had rectal cancer (50%). Seven patients had liver (6 pts) or lung (1 patient) metastases, only. Thirteen patients had extrahepatic, non-lung metastases. Further patient characteristics are shown in Table 1.

**Table 1:** Patients characteristics

**Characteristics**	**n (%)**	**DL1**	DL2*	**DL3**
		**n**	**n**	**n**
**Total patient number**	**20 (100)**	**7**	**7**	**6**
**Gender**				
Male	15 (75)	4	6	5
Female	5 (25)	3	1	1
**Age**				
Median Age – years (range)	59 (34–72 )	57 (34–72)	64 (43–67)	57 (44–69)
**ECOG PS**				
ECOG PS 0	15 (75)	5	7	3
**Primary tumor site**				
Colon	10 (50)	4	5	1
Rectum	10 (50)	3	2	5
**Previous adjuvant therapy**				
None	17 (85)	4	7	6
5-FU (infusional)	3 (15)	3	0	0
5-FU (bolus)	1 (5)	1	0	0
Oxaliplatin	2 (10)	2	0	0
**Number of metastatic sites**				
Median number (range)	1.75 (1–3)	2.1 (1–3)	1.7 (1–3)	1.3 (1–2)
**KRAS status (retrospective)**				
Wild type	14 (70)	6	3	5
Mutant	6 (30)	1	4	1

*One further patient in this dose level was excluded from all analyses owing to i.v. port-catheter dysfunction during the first dose.

### Treatment, DLT and determination of MTD

#### Dose escalation

A minimum of three enrolled patients per dose level was planned. One patient in dose level 1 (DL1, irinotecan 95 mg/m^2^) developed neutropenia grade 4, five further patients in DL1 had no DLTs. In the second dose level (irinotecan 125 mg/m^2^), one patient experienced diarrhoea grade 3 that was regarded as DLT. Five additional patients in DL 2 had no DLTs. Two patients had to be replaced in the first two dose levels, one patient with traumatic pelvic fracture (DL1), and one patient with a late postoperative complication of adhesive ileus (DL2).

In dose level 3 (irinotecan 165 mg/m^2^), three patients experienced a DLT (one patient with diarrhoea grade 3 and two patients with neutropenia grade 4). Therefore, further dose escalation was terminated and 125 mg/m^2^ irinotecan in combination with oxaliplatin, 5-FU/FA and cetuximab was recommended as the dose for further evaluation.

#### Toxicity

Twenty patients were evaluable for toxicity. No therapy associated death was observed. The most common grade ≥3 toxicities were neutropenia (40%), diarrhoea (25%) and acne-like rash (15%). An overview of the toxicities is shown in Table 2.

**Table 2:** Toxicity

**Toxicity**	**All DL n (%)**			**DL1 (n)**		**DL2 (n)**		**DL3 (n)**	
	**n = 20**			**n = 7**		**n = 7**		**n = 6**	
**Grade**	**All**	**Gr. 3**	**Gr. 4**	**All**	**Gr. 3/4**	**All**	**Gr. 3/4**	**All**	**Gr. 3/4**
**Hematologic**									
Neutropenia	14 (70)	3 (15)	5 (25)	5	2	3	2	6	4
Thrombopenia	11 (55)	1 ( 5)	0 (0)	4	1	4	0	3	0
Anaemia	10 (50)	0 (0)	0 (0)	3	0	4	0	3	0
**Gastrointestinal**									
Diarrhoea	15 (75)	5 (25)	0 (0)	6	1	4	2^*^	5	2^*^
Nausea	13 (65)	0 (0)	0 (0)	7	0	4	0	2	0
Vomiting	6 (30)	1 (5)	0 (0)	5	1	1	0	0	0
Stomatitis	14 (70)	0 (0)	0/ 0	6	0	3	0	5	0
Other Mucositis	5 (25)	0 (0)	0 (0)	1	0	1	0	3	0
Bowel Obstruction	1 (5)	0 (0)	1 (5)	0	0	0	1	0	0
**General disorders**									
Fatigue	16 (80)	0 (0)	0 (0)	5	0	5	0	6	0
Skin toxicity	18 (90)	3 (15)	0 (0)	6	1	6	2	6	0
Paronychia	6 (30)	1 (5)	0 (0)	0	0	2	0	4	1

*One patient fulfilling DLT criteria.

#### Treatment duration

The median number of cycles received was 7.5 for DL1, 9.7 for DL2 and 12 for DL3. Two patients received only one cycle of treatment, one patient owing to a late postoperative ileus (DL 2) and one patient due to a traumatic pelvic fracture (DL 1). Eight out of the 20 patients completed 12 cycles (one patient in DL1 and DL2, respectively, and all six patients in DL3). Two patients stopped study therapy for surgery (after 8 and 10 cycles, DL 1 and 2 respectively). Treatment was discontinued due to: unacceptable toxicity in three patients (one patient with diabetic foot ulcer, one patient with skin toxicity (paronychia and polyneuropathy), and one patient with perforation of the transverse colon, all in DL2); withdrawal of consent in three patients (after 6, 8 and 11 cycles respectively, all DL1); complete response of all lesions in one patient, (after 7 cycles, DL1) and a adminstrative mistake in one patient (after 11 cycles, DL2).

#### Dose intensity

**Figure 2:**
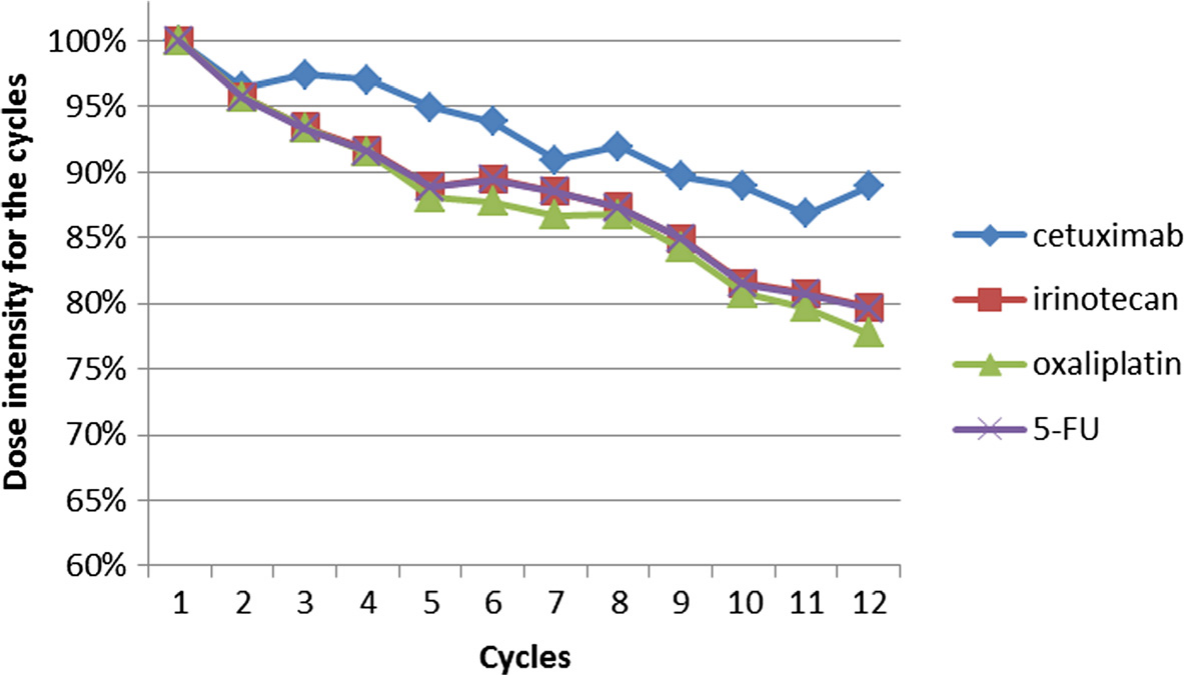
Dose intensity from treatment start according to the cycle.

The dose intensity for the first six cycles was 94%, 89%, 88% and 89% for cetuximab, irinotecan, oxaliplatin and 5-FU. The dose intensity from treatment start over the time is shown in Figure 2.

### Efficacy

#### Tumour response

**Figure 3:**
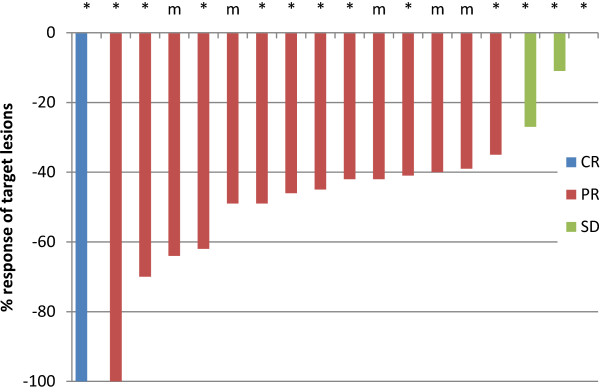
**Best response of target lesions by patient, regardless of k-ras status (evaluable patients). **CR – complete response, PR – partial response, SD – stable disease, m - k-ras mutant, * - k-ras wildtype Two patients (one patient with k-ras wt and one patient with k-ras mutant tumour) were not evaluable for response as they were excluded from the study after cycle one without response evaluation. One patient with stable disease had no change in tumor size.

In all treated patients, the overall confirmed response rate was 75% (15/20 patients, 95% CI 51-91%). Fourteen patients (70%, 95% CI 48-86%) had a partial response (PR) and one patient (5%) had a complete response (CR). Three patients (15%, 95% CI 4-37%) had stable disease (SD). There was no patient with progressive disease as best response; the two replaced patients (with traumatic pelvic fracture and with mechanical bowel obstruction) were not evaluable for response. Best response was reached after a median of 3.1 months. The responses according to cohorts and the waterfall plot are shown in Table 3 and Figure 3.

**Table 3:** Response to treatment

	**Total (n)**	**DL1 (n)**	**DL2 (n)**	**DL3 (n)**
	**n = 20**	**n = 7**	**n = 7**	**n = 6**
**Response**				
Complete Remission	1	1	0	0
Partial Remission	14	2	6	6
Stable Disease	3	3	0	0
Not evaluable	2	1	1	0

In three out of twenty patients study treatment was followed by metastasectomy after 8, 10 and 12 cycles.

K-ras mutational status was determined in all 20 patients. In 14 patients with k-ras wild type tumours, one patient had complete response and nine PR (response rate 72% [95% CI 42-92%]), three patients stable disease, one was not evaluable. Out of six k-ras mutant patients, five were evaluable for response. All five patients had a partial response.

Six out of seven patients with metastases limited to the liver or lung responded to treatment (86%) compared to 9/13 patients with extrahepatic, non-lung metastases (69%).

#### Progression free and overall survival

**Figure 4:**
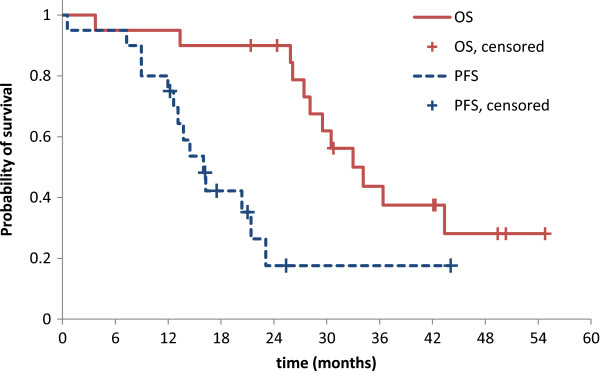
Overall survival (OS) and Progression free survival (PFS).

Twenty patients were evaluable for survival analysis. At the time of analysis, eight patients (40%) were alive, seven with disease progression and one without progression.Median progression free survival (PFS) was 16.0 (95%-CI 12.6-19.4) months, overall survival (OS) was 33.0 (95%-CI 26.2-39.8) months (Figure 4). Eighteen patients (90%) received second line treatment which was heterogeneous, consisting mostly of antibody or doublet combinations (bevacizumab/FOLFIRI - 6 pts, bevacizumab/5-FU 3 pts, FOLFIRI-like schedules 6 pts, cetuximab/FOLFOX – 2 pts, cetuximab/FOLFIRI – 1 patient).

The median OS did not differ between patients with metastases confined to liver and lung (33.0 months, 95% CI 25.6 – 39.4 months) and patients with other metastases (34.1 months, 95% CI 23.9 – 44.3 months, p = 0.6). The median progression free survival was 21.4 months [95% CI: 10.3 – 32.5] and 14.5 months [95% CI: 10.76 – 18.2, p = 0.99] in patients with liver/lung metastases and patients with extrahepatic, non-lung metastases.

## Discussion

Adding oxaliplatin or cetuximab to FOLFIRI resulted in higher RR, longer OS and higher rates of liver resection [6,8]. To combine both approaches might be an effective way to intensify treatment in selected patients with good performance status and k-ras wild type tumours. However, both combinations were associated with increased toxicity compared to FOLFIRI alone. To prepare further trials, the aim of this phase I trial was to determine the MTD of the chemotherapy combination of cetuximab, irinotecan, oxaliplatin and 5-FU/FA for future studies in patients with non-resectable liver metastases.

In our study, we have shown that it is feasible to administer cetuximab in combination with oxaliplatin, 5-FU/FA and irinotecan. However, diarrhoea and neutropenia limited the maximum dose of irinotecan which is recommended for further trials to 125 mg/m^2^, lower than in the FOLFOXIRI schedule (165 mg/m^2^) without cetuximab [6].

Our findings of increased toxicity are in line with the POCHER trial investigating the same drugs in a chronomodulated schedule requiring a dose reduction during the trial [12]. A French trial investigating cetuximab and FOLFIRINOX did not reduce the chemotherapy (180 mg/m^2^ irinotecan, 85 mg/m^2^ oxaliplatin and 5-FU/FA) but reported a diarrhoea grade 3/4 rate of 52%, and a neutropenia grade 3/4 rate of 38% [13].

The confirmed response rate of 75% (irrespective of the k-ras status) is promising and - in the historical comparison - numerically higher than FOLFOXIRI (response rate of 66% [6]) or cetuximab/FOLFIRI (confirmed response rates 57.3% in k-ras wild type and 39.7% in k-ras mutant patients [8]). However, the low patient number and the subsequently large confidence interval in our study do not allow any final conclusion, especially for subgroup analysis, i.e. regarding the unexpected finding that the response was slightly higher in k-ras exon 2 mutant patients or patients or the known phenomenon of numerically higher response rates in patients with metastases in liver or lung, only.

In this trial that did not select patients with potentially resectable metastases but included a relatively high proportion of patients with more than one metastatic site (55%), three patients were resected for metastases and one patient stopped chemotherapy prematurely because of an early complete response. The overall survival belongs to the longest reported in clinical trials for metastatic colorectal cancer that were not conducted in the setting of neoadjuvant therapy. Patient selection, the high number of patients with second line treatment (90%) and the concept of an intensive induction treatment may have contributed to the favourable outcome.

The short time until best response of 3.1 months might be important for neoadjuvant chemotherapy in potentially resectable metastases, as a higher number of chemotherapy cycles is associated with higher perioperative morbidity [14]. Even though the distribution of PS is comparable to other trials investigating intensive chemotherapy combinations [6], we do not recommend this schedule for patients with an impaired PS, considering, that 75% of the patients in our trial had a WHO PS 0. Our regimen might be a treatment option for patients with potentially resectable liver metastases, in whom the chance of a curative resection outweighs the risk of the high toxicity of such an intensive regimen, and also in other patients with a high need for intensive treatment [15]. The ongoing CELIM2 study is investigating this chemotherapy schedule in patients with k-ras wild-type liver metastases (NCT01802645).

## Conclusions

In conclusion, this phase I trial was able to demonstrate that this intensive combination of cetuximab, oxaliplatin, 5-FU/FA and irinotecan in mCRC is feasible and has an acceptable toxicity profile in patients with a good PS. In these patients, the observed clinical activity with a confirmed response rate of 75% is promising and might be interesting i.e. in neoadjuvant treatment situations.

## Competing interests

The study was supported by an educational grants of Merck Pharma Deutschland GmbH and Pfizer Pharma Deutschland GmbH.

Gunnar Folprecht received research funding (Merck KGaA), honoraries for ad-hoc advisory boards (Merck KGaA, Genentech, Sanofi-Aventis, Lilly, Bayer, Bristol-Myers Squibb) and lecture fees (Merck KGaA, Genentech, Sanofi-Aventis, Lilly).

## Authors’ contributions

GF has designed the trial, was involved in acquiring, analysing, interpretation of the data and manuscript writing. SH has been involved in data analysis and manuscript writing. KS participated in protocol writing as well as data analysis. TT, JSW and GE were involved in acquiring and interpretation of the data. All authors read and approved the final manuscript.

## Pre-publication history

The pre-publication history for this paper can be accessed here:

http://www.biomedcentral.com/1471-2407/14/521/prepub
